# Dietary Behavior Clustering and Cardiovascular Risk Markers in a Large Population Cohort

**DOI:** 10.3390/nu18030533

**Published:** 2026-02-05

**Authors:** Mauro Lombardo, Giovanni Aulisa, Fares M. S. Muthanna, Sercan Karav, Sara Baldelli, Gianluca Tripodi, Gilda Aiello

**Affiliations:** 1 Department for the Promotion of Human Science and Quality of Life, San Raffaele Open University, Via di Val Cannuta, 247, 00166 Rome, Italy; aulisadietista@gmail.com (G.A.); sara.baldelli@uniroma5.it (S.B.); gianluca.tripodi@uniroma5.it (G.T.); gilda.aiello@uniroma5.it (G.A.); 2 Pharmacy Department, Faculty of Medicine and Health Sciences, University of Science and Technology-Aden, Alshaab Street, Enmaa City 22003, Yemen; f.mothana@aden.ust.edu; 3 Department of Molecular Biology and Genetics, Çanakkale Onsekiz Mart University, Canakkale 17000, Türkiye; sercankarav@comu.edu.tr; 4 IRCCS San Raffaele Roma, 00166 Rome, Italy

**Keywords:** eating behavior profiles, behavioral nutrition, cardiometabolic health, dietary habits, body composition, food choice, nutritional assessment

## Abstract

Background: Eating habits influence cardiometabolic health alongside traditional dietary measures. However, the links between dietary patterns, body composition, and heart-healthy food preferences remain under-explored in large cohorts. Methods: In this cross-sectional study, 2461 adults (aged 18 to 75 years) completed an online survey on eating behaviors, food preferences, and lifestyle. Principal component analysis (PCA) of seven behaviors identified dietary profiles. A heart-healthy diet score (range −2 to 10; higher = greater preference for fruit, vegetables, legumes, fish, and less meat/processed meat) was derived from these food preferences. ANOVA and adjusted regressions linked the profiles to BMI, fat mass, waist circumference, and diet score. Results: Four profiles emerged: structured, social, irregular, and disordered eaters. Structured eaters had the lowest BMI (26.8 ± 5.1 kg/m^2^), lowest fat mass (28.9 ± 9.4%), and highest dietary score (4.73 ± 2.0). Disorganized eaters had the highest BMI (29.0 ± 5.5 kg/m^2^), the highest fat mass (31.2 ± 8.8%) and the lowest score (3.93 ± 2.0); all *p* < 0.05. Dose–response analyses confirmed that greater disordered eating (PCA1) was associated with worse outcomes. Conclusions: Dietary profiles are associated with body composition and cardioprotective preferences. Behavioral assessment could refine the identification of cardiometabolic risk and personalize nutrition.

## 1. Introduction

Cardiovascular disease (CVD) remains the leading cause of death globally, accounting for nearly one-third of all deaths each year [1]. In addition to traditional risk factors, such as hypertension, dyslipidemia, and smoking, emerging research highlights the importance of lifestyle-related behavioral determinants, including dietary patterns and eating behaviors, in the prevention and management of CVD [2,3].

Although numerous studies support the role of individual nutrients and food groups in cardiometabolic health, nutritional behavioral models highlight that eating behaviors, such as meal timing, speed, emotional cues and phenotyping through data-based clustering, independently influence diet quality and risk beyond simple calorie intake. Phenotyping eating behaviors, often through principal component analysis, reveals profiles (e.g., structured vs. disordered) that predict outcomes more holistically than individual traits. Recent meta-analyses confirm inverse associations between prudent/healthy dietary patterns and coronary heart disease risk [4], while fast eating speed is linked to hypertriglyceridemia and metabolic syndrome components [5].

Recent evidence indicates that eating behaviors such as breakfast skipping, irregular meal timing, and late-night eating are linked to adverse body composition and early cardiometabolic risk, even in individuals without overt obesity [6,7,8]. However, large-scale studies integrating eating behavior phenotyping with body composition and cardioprotective dietary preferences remain scarce. No previous work has grouped self-reported behaviors into profiles and linked them to both anthropometric phenotypes and preference-based dietary scores in diverse adults. This cross-sectional study fills this gap by using PCA to identify profiles and test their associations with BMI, fat mass, waist circumference, and a new cardioprotective score, providing useful information for personalized nutrition.

## 2. Materials and Methods

### 2.1. Subjects

Participants were recruited between January 2024 and March 2025 at a specialized obesity center in Rome through a digital survey disseminated via online platforms and social media. This dual recruitment strategy was implemented to increase the diversity of the sample in terms of geography and sociodemographic background. A total of 2750 individuals accessed and completed the online questionnaire. Eligibility criteria required participants to be between the ages of 18 and 75 years, to have completed the entire survey, and to have provided informed consent. Exclusion criteria included a self-reported diagnosis of cardiovascular or metabolic disease, current pregnancy or breastfeeding, recent participation in a medically supervised weight loss program, or incomplete or inconsistent responses. As a result, 52 participants were excluded because of cardiovascular or metabolic disease, 27 because of pregnancy or lactation, 25 because of recent participation in weight loss programs, and 185 because of incomplete or inconsistent data. The final analytical sample included 2461 adults. The sample size was defined to ensure adequate statistical power to detect group differences in primary outcomes. A priori power analysis indicated that a sample size of at least 2000 participants would yield greater than 90% power to detect small to moderate effect sizes (Cohen’s d = 0.3) at a two-sided significance level of 0.05. The study was approved by the Lazio Area 5 Territorial Ethics Committee (Approval Code: N.57/SR/23, 7 November 2023) and conducted in accordance with the principles of the Declaration of Helsinki. This study was registered prospectively on ClinicalTrials.gov (identifier: NCT06654674).

### 2.2. Questionnaire

The questionnaire, developed on the basis of previous instruments relating to eating behavior and food preferences but not formally validated, was designed to be completed independently at a distance (25–30 min). Its strengths include brevity and ecological validity for large-scale screening; limitations (unvalidated status and self-assessment bias) are discussed in Section 4. Completion ensured anonymity through electronic consent. The questionnaire was designed to be completed remotely and self-administered and was accessible via computer or mobile devices. Completion time was approximately 25–30 min. After accessing the platform, participants provided informed consent electronically before beginning the survey. No identifying data were collected, ensuring complete anonymity of responses. The questionnaire was developed based on previous instruments used in studies of eating behavior and food preferences, and, although it was not formally validated, it maintained structural consistency with existing instruments [9]. It consisted of four main sections. The first part explored eating behaviors, including timing and frequency of meals and subjective hunger throughout the day. Participants were asked whether they usually skip meals, eat quickly, consume food while distracted or not seated at the table, or experience episodes of uncontrolled eating without hunger. Night feeding episodes were also recorded. Section 2 focused on food preferences. Participants were asked to indicate their liking for a list of common foods using a simple binary format (“Yes”/”No”). Binary responses were preferred over frequency-based measures for three reasons: (1) simplicity/scalability for online implementation; (2) focus on liking (a validated indicator of intake) versus frequency, which is influenced by recall; and (3) alignment with previous diet quality scores derived from liking that show strong cardiometabolic associations. This approach prioritizes feasibility while capturing qualitative alignment of diet. The list included animal products (e.g., meat, fish, eggs, milk, yogurt, and cheese), plant-based options (e.g., legumes, tofu, nuts, and soy milk), and fiber-rich foods (e.g., vegetables, fruits, grains, and whole grains). The responses were then used to construct a cardiovascular dietary score based on the number of favorable versus unfavorable choices. In Section 5, participants were asked about physical activity. Participants stated whether they practiced sports or structured exercise and, if so, the number of hours per week (<5, 5–10, or >10 h), as well as the time of day and type of sport they practiced.

#### Reproducibility and Internal Consistency

Test–retest reliability (*n* = 50, 2-week interval) showed good stability for behavioral items (ICC = 0.72–0.85) and moderate stability for preferences (ICC = 0.65). Internal consistency among the seven PCA behaviors was acceptable (Cronbach’s α = 0.68); the cardioprotective score showed fair consistency (α = 0.61), in line with the heterogeneous food categories.

### 2.3. Body Composition

Body composition was assessed at the medical center using standardized protocols. Participants arrived after fasting (minimum 8 h), wearing only light undergarments. Body weight was measured with a calibrated TANITA BC-420 MA bioimpedance scale (TANITA Corporation, Tokyo, Japan), with an accuracy of 100 g [10,11]. Height was measured with a wall stadiometer while the participant stood with the head aligned with the Frankfurt horizontal plane. BMI was calculated as body weight divided by height squared (kg/m^2^). Abdominal circumference (AC) was measured at the midpoint between the lowest rib and the iliac crest, with the subject standing and breathing normally. Fat mass (FM) and fat-free mass (FFM) were obtained using the same TANITA BC-420 MA bioimpedance device. To minimize variations due to hydration or physical exertion, measurements were taken at least three hours after waking up, three hours after eating, and 12 h after any strenuous physical activity. Women were asked not to schedule measurements during menstruation. Each body composition variable was assessed twice, and the mean value was used for analysis.

### 2.4. Identification of Behavioral Eating Profiles via Principal Component Analysis

PCA was applied to responses from 7 behavioral questions with established cardiometabolic associations: hunger timing, meal skipping, distracted eating, eating speed, social eating, uncontrolled eating, and night eating (Table 1). Categorical variables were coded by one-hot encoding and standardized before PCA was applied. The subjects were divided into four groups, which were interpreted according to the prevailing behavioral characteristics: structured eaters, social eaters, irregular eaters, and disordered eaters. The main characteristics of each group derived from the PCA are summarized in Table 1. The table shows the most frequent response for each of the six behavioral variables within each group, illustrating the distinct eating behavior profiles that emerged from the classification. PCA details and the 4-cluster determination methodology are reported in Supplementary Table S1.

### 2.5. Cardiovascular-Protective Diet Score

To explore the relationship between behavioral food profiles and dietary patterns related to cardiovascular risk, we constructed a protective dietary score for CVD based on self-reported food preferences. Participants responded “Yes” or “No” to liking specific food categories. Sixteen foods were considered, but only those with a sufficient response rate and relevant to cardiovascular health were included in the score. Protective items included fruits, raw and cooked vegetables, legumes, cereals, whole grains, tofu, nuts, soy drinks, and fish. Unfavorable items included meat and processed meats. Each “Yes” response to a protective food contributed 1 point, while each “Yes” response to an unfavorable food subtracted 1 point. “Cow’s milk” was considered neutral (0 points). Blank or invalid responses were not included in the score calculation. The final score ranged from −2 to +10, with higher values indicating greater adherence to a cardioprotective preference profile. Mean CVD diet scores were calculated for each behavioral group identified by PCA [12]. This preference-based scoring approach aligns with prior liking-based diet quality indices that correlate with cardiometabolic outcomes [13,14]. While not formally validated against clinical endpoints or dietary intake, the score provides a scalable, exploratory measure of alignment with cardioprotective food recommendations.

### 2.6. Statistical Analysis

Continuous variables were expressed as means ± standard deviations and compared between males and females by independent *t*-tests. Categorical variables were expressed as percentages and compared by chi-square test. Comparisons between the four PCA-derived eating behavior groups were conducted using one-way analysis of variance (ANOVA) for continuous variables and chi-square tests for categorical variables (*p* < 0.05). As primary outcomes were pre-specified, no multiple testing correction was applied; exploratory analyses were interpreted cautiously. Exact *p*-values are reported in tables for transparency. For body composition outcomes, multivariable linear regression models assessed associations with eating behavior groups, adjusting for sex, age, and physical activity. The models demonstrated adequate fit with no multicollinearity. Additional dose–response analyses used PCA1 scores as continuous predictors. All analyses used Python 3.11 (pandas, numpy, scikit-learn, and statsmodels).

## 3. Results

A total of 2461 individuals (1010 males and 1451 females) were analyzed (Table 2). Females were slightly older than males (*p* < 0.001). Males had higher muscle mass/BMR; females higher fat % (Table 2). Physical activity levels were similar between sexes (*p* = 0.12).

PCA behavioral groups showed clear outcome gradients (Table 3): Structured eaters had the lowest BMI/fat mass and highest activity vs. disordered eaters (highest BMI/fat mass). Social/irregular groups were intermediate; all differences were significant (*p* < 0.001) except sex (*p* = 0.094).

Adjusted analyses confirmed that structured eaters had significantly better body composition than disordered eaters across all metrics (Table 4). Social/irregular groups showed intermediate risk profiles.

**Figure 1:**
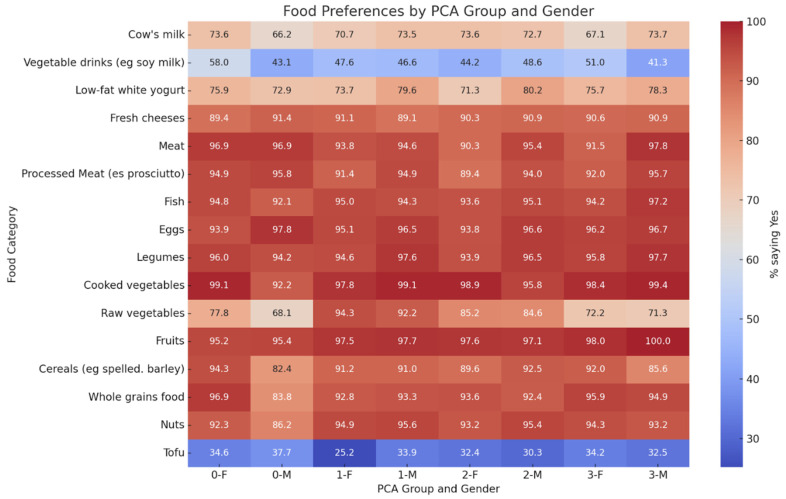
Food preferences by PCA behavioral group and sex. Heat map of food preferences by PCA group/gender. No statistically significant differences were found between groups according to chi-square tests for the following food categories: cow’s milk (*p* = 0.2561), vegetable beverages (*p* = 0.4816), low-fat white yogurt (*p* = 0.5316), fresh cheese (*p* = 0.2144), meat (*p* = 0.4295), processed meat (*p* = 0.2572), fish (*p* = 0.1654), eggs (*p* = 0.2063), legumes (*p* = 0.1146), cooked vegetables (*p* = 0.2615), raw vegetables (*p* = 0.2965), fruits (*p* = 0.1083), grains (*p* = 0.4142), whole grains (*p* = 0.3997), nuts (*p* = 0.0796), and tofu (*p* = 0.2724).

Food preferences showed non-significant trends toward healthier choices in structured and social eaters (Figure 1), with no statistically significant differences across individual food categories.

**Figure 2:**
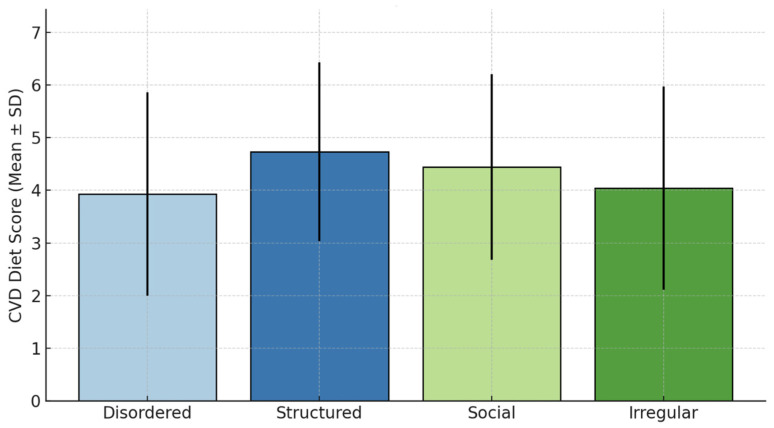
Cardioprotective diet score by PCA behavioral group. Mean ± SD. Score based on food preferences (−2 to +10 range). ANOVA *p* < 0.001 across groups.

Cardioprotective diet score demonstrated a significant gradient across groups (Figure 2; ANOVA *p* < 0.001)—structured eaters (highest), followed by social, irregular, and disordered eaters (lowest)—mirroring body composition patterns.

## 4. Discussion

This study demonstrates that PCA-derived dietary behavioral profiles provide a holistic phenotyping of temporal, social, and control-related dietary traits that are linked to adiposity and cardiometabolic risk [15,16,17,18]. By integrating these behavioral patterns with a composite cardioprotective dietary score, we extend previous work from individual behaviors to multidimensional profiles that better reflect real-world eating habits. To our knowledge, this is one of the first studies to directly link such behavioral constructs with a preference-based cardioprotective index. The association between dietary behavioral profiles and food preferences supports the idea that how people eat influences what they choose to eat: structured and social profiles were more consistent with cardioprotective foods, while disordered patterns were less so, consistent with previous evidence related to uninhibited/emotional eating and high-energy choices [19,20]. Our preference-based CVD score offers an innovative and scalable approach compared to traditional intake frequency metrics, facilitating risk stratification in clinical practice.

Food preference patterns showed no significant differences across individual categories, despite the composite cardioprotective diet score gradient. This suggests that eating behavior profiles influence overall dietary patterning more than isolated preferences. Possible explanations include the fact that (1) the binary preference format lacks sensitivity for subtle differences, (2) the fact that structured eaters better translate preferences into consistent choices, and (3) the fact that composite scores are superior for detecting behavioral–diet links [21]. Thus, while preferences/behaviors show partial decoupling at the item level, our findings validate a composite preference-based assessment for cardiometabolic profiling.

In addition to categorical grouping, we also explored the relationship between the degree of behavioral disorganization and body composition through a dose–response analysis using PCA1 as a continuous predictor. The results revealed a consistent and statistically significant association: for each unit increase in PCA1 score—reflecting more disordered eating behaviors—there was a corresponding increase in BMI, percent fat mass, and abdominal circumference, as well as a decrease in fat-free mass. These associations remained robust after adjusting for sex, age, and physical activity. This continuous approach adds analytical strength to the categorical results and supports the presence of a gradient effect, in which small increases in disordered eating traits are associated with progressively less favorable body composition. These findings are in line with previous research linking uninhibited eating scales and cognitive restriction to anthropometric results, but provide a more integrative, model-based view of eating behavior [22,23].

These behavioral gradients likely operate through integrated physiological pathways. Structured profiles promote circadian alignment of hepatic and adipose clocks, improving insulin sensitivity and mitigating ectopic fat accumulation. Morning hunger patterns stabilize ghrelin–leptin oscillations, suppressing compensatory hyperphagia and supporting energy homeostasis. Increased prefrontal inhibition simultaneously attenuates stress-induced emotional eating. Disordered patterns disrupt these systems through hypothalamic desynchronization and impaired satiety signaling, producing dose-dependent increases in adiposity observed in PCA1 scores. The observed associations between disordered eating behaviors and higher fat mass, abdominal adiposity, and lower fat-free mass are consistent with evidence showing that unfavorable lifestyle habits can promote metabolically adverse body composition even in individuals without overt obesity [24]. Previous studies [25,26] in adult populations have highlighted that irregular meal patterns and skipping breakfast are associated with increased visceral fat accumulation and reduced lean mass, supporting the notion that behavioral factors may contribute to subclinical cardiometabolic risk.

From a clinical perspective, identifying behavioral food profiles may offer an accessible and low-cost means of stratifying individuals according to their nutritional risk before the onset of overt cardiometabolic disease [27]. Since the behavioral variables used in our analysis are simple, non-invasive and easily detectable via questionnaires, they could be incorporated into routine screening alongside BMI, waist circumference and other lifestyle indicators in primary care and health prevention programs. Similarly to existing diet-based risk prediction models, behavioral scores could complement traditional clinical metrics and improve the early identification of individuals at risk [28]. Integration into digital health platforms could further improve scalability and implementation. Incorporating these measures into electronic health records, patient portals, or mobile applications could enable automated assessment, longitudinal monitoring, and personalized feedback. For example, individuals with more disorganized profiles, previously linked to higher cardiometabolic risk and unhealthy behaviors, could receive targeted suggestions aimed at improving meal regularity, planning, or appetite awareness, while structured profiles could benefit from reinforcement strategies [29]. Such approaches can support continuous risk stratification and facilitate behavior-focused interventions that complement traditional calorie- or nutrient-based counseling. Notably, structured eating behaviors identified in the present study share key features with lifestyle patterns previously associated with more favorable body composition, including regular breakfast consumption and more stable meal structure. Evidence from adult cohorts suggests that regular breakfast intake and higher meal regularity are linked to lower fat mass indices, higher phase angle, and more favorable lean mass distribution, highlighting potential mechanisms through which behavioral eating profiles may influence cardiometabolic health [30].

This study has several limitations. First, the cross-sectional design precludes causal inferences and temporal relationships between dietary behavior profiles and body composition outcomes. Second, all data were self-reported, including food preferences, physical activity, and behavioral patterns, which could be subject to recall bias, social desirability bias, and inherent inaccuracies of non-validated questionnaires. Third, although the questionnaire was developed based on previously validated instruments [9], it lacks formal validation against objective measures or clinical endpoints, which could introduce measurement bias. Fourth, the cardioprotective diet score, although aligned with evidence-based dietary recommendations, has not been validated against dietary intake or strict cardiometabolic endpoints. Fifth, the absence of clinical measures such as blood pressure, fasting glucose, lipid profiles, or inflammatory markers limits the direct assessment of cardiovascular risk beyond body composition proxies. Sixth, bioelectrical impedance analysis, while practical for large-scale assessments, is sensitive to hydration status and less precise than gold-standard methods such as DXA. Finally, residual confounding may persist despite multivariate adjustment. However, these limitations are offset by the large sample size, consistency across multiple outcomes, dose–response relationships, and the novel integration of behavioral phenotype with cardioprotective preferences, providing solid evidence for hypothesis generation and future prospective validation.

## 5. Conclusions

In this large population-based study, we identified distinct behavioral eating profiles using principal component analysis and demonstrated their strong association with body composition and cardiovascular-related eating patterns. Individuals with structured and mindful eating behaviors showed healthier anthropometric parameters and greater adherence to cardioprotective food preferences, whereas disordered eaters showed the most unfavorable profiles. These findings suggest that eating behaviors—beyond nutrient intake—may serve as early and practical indicators of cardiometabolic risk. Evaluation of behavioral patterns in routine clinical settings could offer a valuable and scalable strategy for tailoring nutritional interventions to promote long-term cardiovascular health.

## Figures and Tables

**Table 1:** Predominant behavioral responses by PCA-derived eating groups. Modal responses to 7 behavioral questions across four PCA clusters (structured, social, irregular, disordered eaters).

PCA Group	When Hungry?	Miss Meals?	Distracted Eating?	Eat Fast?	Eat Alone/Together?	Uncontrolled Eating?	Night Eating?
Disordered	Before Dinner	Yes	Yes	Yes	Often Together	Often (>1/week)	Rarely (once/month)
Structured	Morning	No	No	Yes	Often Together	Rarely (once/month)	Never
Social	Before Dinner	No	Yes	Yes	Often Together	Often (>1/week)	Never
Irregular	Before Dinner	Yes	Yes	Yes	Often Together	Often (>1/week)	Never

**Table 2:** Sample characteristics by sex (mean ± SD or %). BMI, body mass index; FM, fat mass; AC, abdominal circumference; FFM, fat-free mass; BMR, basal metabolic rate. Differences by unpaired *t*-tests/χ^2^ (*p* < 0.001).

Variable		Total (n. 2461)	Males (n. 1010)	Females (n. 1451)	*p*-Value (M vs. F)
Age		40.9 ± 13.1	39.6 ± 12.8	41.8 ± 13.2	<0.001
Smoker (%)	Yes	24.1	24.5	23.5	0.1000
Weight (kg)		78.8 ± 17.6	88.6 ± 17.2	72.1 ± 14.5	<0.001
BMI (kg/m^2^)		27.7 ± 5.3	28.5 ± 5.1	27.2 ± 5.3	<0.001
FM (kg)		24.3 ± 10.8	22.6 ± 10.9	25.5 ± 10.6	<0.001
FM (%)		30.1 ± 9.2	24.4 ± 7.6	34.1 ± 8.0	<0.001
AC (cm)		96.0 ± 14.2	100.5 ± 14.2	92.9 ± 13.4	<0.001
FFM (kg)		51.9 ± 11.3	62.8 ± 8.2	44.2 ± 5.2	<0.001
FFM (%)		66.5 ± 8.8	71.9 ± 7.3	62.6 ± 7.7	<0.001
Water (kg)		38.4 ± 8.5	46.5 ± 6.3	32.8 ± 4.3	<0.001
BMR (kcal)		1638.8 ± 342.0	1951.1 ± 274.8	1421.4 ± 175.1	<0.001
Income	<€20,000	15.2	17.1	12.5	<0.001
Income	>€60,000	2.9	2.8	3.0	<0.001
Income	€20,000–€40,000	68.7	67.5	70.4	<0.001
Income	€40,000–€60,000	13.2	12.6	14.1	<0.001
Sport (%, yes)		53.6	48.9	60.4	<0.001
Sport weekly hours	<5	68.0	76.0	56.6	<0.001
	5–10	27.8	21.2	37.2	<0.001
	>10	3.3	1.9	5.2	<0.001

**Table 3:** Characteristics of PCA-derived behavioral eating groups (mean ± SD or %). BMI, body mass index; FM, fat mass; FFM, fat-free mass; AC, abdominal circumference. One-way ANOVA/χ^2^; all *p* < 0.001 except sex (*p* = 0.094).

PCA Group	Gender	Age	Smokers	BMI	FM (%)	FFM (%)	AC	Bowel Movements Weekly	Income < €20,000/Year (%)	Do You Play a Sport?
Disordered	M: 101 (47.4%), F: 112 (52.6%)	37.2 ± 12.3	35.7%	29.0 ± 5.5	31.2 ± 8.8	65.3 ± 8.6	99.9 ± 14.7	6.1 ± 1.7	20.2%	51.2%
Structured	M: 369 (41.3%), F: 524 (58.7%)	43.1 ± 13.9	22.7%	26.8 ± 5.1	28.9 ± 9.4	67.7 ± 9.2	93.9 ± 13.7	6.2 ± 1.5	13.5%	58.5%
Social	M: 346 (38.4%), F: 556 (61.6%)	41.1 ± 12.4	22.6%	28.0 ± 5.2	30.9 ± 9.0	65.7 ± 8.6	96.3 ± 13.8	6.1 ± 1.6	13.7%	51.9%
Irregular	M: 197 (41.7%), F: 274 (58.1%)	38.8 ± 12.7	23.7%	28.3 ± 5.4	30.8 ± 9.0	65.9 ± 8.6	97.5 ± 15.0	6.1 ± 1.7	18.6%	48.9%

**Table 4:** Multivariable-adjusted body composition differences by PCA behavioral group vs. disordered reference. Linear regression coefficients (β) controlling for age, sex, physical activity. BMI, body mass index; FM, fat mass; FFM, fat-free mass; AC, abdominal circumference; BMR, basal metabolic rate.

PCA Group Comparison vs. Reference	BMI	FM (%)	FFM (%)	AC	BMR
C (PCA_group) [T.1]	−2.5 (*p* < 0.001)	−3.8 (*p* < 0.001)	3.82 (*p* < 0.001)	−6.83 (*p* < 0.001)	−87.34 (*p* < 0.001)
C (PCA_group) [T.2]	−1.24 (*p* = 0.0007)	−1.88 (*p* = 0.0003)	1.99 (*p* = 0.0001)	−4.04 (*p* < 0.001)	−40.68 (*p* = 0.0146)
C (PCA_group) [T.3]	−0.83 (*p* = 0.0347)	−1.41 (*p* = 0.0125)	1.56 (*p* = 0.0044)	−2.76 (*p* = 0.0055)	−28.91 (*p* = 0.1078)

## Data Availability

The datasets generated and analyzed during the current study are available from the corresponding author upon reasonable request. All shared data will be anonymized to protect participant confidentiality.

## Supplementary Materials

The following supporting information can be downloaded at: https://www.mdpi.com/article/10.3390/nu18030533/s1, Table S1: Principal component analysis results—behavioral questions and group characteristics.
