# Supplementary material for: Dietary Behavior Clustering and Cardiovascular Risk Markers in a Large Population Cohort
Source: Nutrients. 2026 Feb 5;18(3):533. doi: 10.3390/nu18030533 (PMC12899149; doi:10.3390/nu18030533)
Supplement: Supplementary file 1 [file nutrients-18-00533-s001.zip › nutrients-4131950-supplementary.pdf]

Supplementary Table S1. Principal Component Analysis Results - Behavioral Questions and Group Characteristics.

PCA Component	Primary Dominant Behaviors	Secondary Dominant Behaviors	Tertiary Behaviors	Interpretation	Resulting Group
**PC1: Disorganization**	Uncontrolled eating (>1/week)	Fast eating (Yes)	Distracted eating (Yes)	Loss of appetite control; rushed meals	Disordered Eaters
**PC2: Social Eating**	Eating together (Often)	Hunger before dinner (Yes)	Meal skipping (No)	Structured social meals; regular appetite	Social Eaters
**PC3: Structure**	Morning hunger (Yes)	No night eating (Never)	No distracted eating (No)	Regular circadian rhythm; mindful eating	Structured Eaters
**PC4: Irregularity**	Meal skipping (Yes)	Hunger before dinner (Yes)	Night eating (Never to Often)	Erratic meal patterns; inconsistent timing	Irregular Eaters

Methodology Notes

PCA Input: 7 behavioral questions from dietary questionnaire (Table 1)
Categorical Encoding: One-hot encoding applied to all response categories
Standardization: Z-score normalization (scikit-learn, Python 3.11)
Clustering Method: k-means on principal component scores
Optimal k Determination: Elbow method + silhouette analysis (optimal k=4)
Variance Explained: PC1–PC4 account for primary behavioral variation in cohort

Group Definitions (from Table 1 Modal Responses)

Disordered Eaters (PC1 dominant)

- Often uncontrolled eating (>1/week)
- Often eat fast (Yes)
- Often eat distracted (Yes)
- Hunger before dinner
- Often eat together
- Rarely night eating (once/month)

Social Eaters (PC2 dominant)

- Often eat together
- Hunger before dinner
- No meal skipping
- Often eat distracted (Yes)
- Often eat fast (Yes)
- Never uncontrolled eating
- Never night eating

Structured Eaters (PC3 dominant)

- Morning hunger
- No meal skipping
- Often eat fast (Yes)
- Often eat together
- No distracted eating
- Rarely uncontrolled eating (once/month)
- Never night eating

Irregular Eaters (PC4 dominant)

- Hunger before dinner
- Meal skipping (Yes)
- Often eat distracted (Yes)
- Often eat fast (Yes)
- Often eat together
- Often uncontrolled eating (>1/week)
- Never night eating

Statistical Justification

The 7-item behavioral questionnaire was selected based on:

1. **Established cardiometabolic associations -** Each question addresses eating behaviors linked to cardiovascular and metabolic health outcomes
2. **Behavioral independence -** Variables capture distinct eating dimensions (temporal, social, control, speed)
3. **Categorical clarity -** Response options facilitate clear clustering without loss of information
4. **Clinical relevance -** All behaviors have measurable nutrition intervention implications

k-means clustering on PCA scores optimized at k=4 through standard elbow and silhouette criteria, yielding four distinct, interpretable eating behavior phenotypes suitable for downstream outcome analysis.
